# Dental Caries and Salivary Oxidative Stress: Global Scientific Research Landscape

**DOI:** 10.3390/antiox12020330

**Published:** 2023-01-31

**Authors:** Yago Gecy de Sousa Né, Weslley Ferreira Lima, Paulo Fernando Santos Mendes, Daiane Claydes Baia-da-Silva, Leonardo Oliveira Bittencourt, Priscila Cunha Nascimento, Renata Duarte de Souza-Rodrigues, Luiz Renato Paranhos, Paulo Antônio Martins-Júnior, Rafael Rodrigues Lima

**Affiliations:** 1 Laboratory of Functional and Structural Biology, Institute of Biological Sciences, Federal University of Pará, Belém 66075-110, Brazil; 2 Department of Community and Preventive Dentistry, Federal University of Uberlândia, Uberlândia 38408-144, Brazil; 3 Department of Child and Adolescent Oral Health, School of Dentistry, Federal University of Minas Gerais, Belo Horizonte 31270-901, Brazil

**Keywords:** oxidative stress, dental caries, saliva

## Abstract

This study aimed to analyze the research trends on salivary oxidative stress associated with dental caries and to perform bibliometric approaches for existing publications on this association. A search was performed using the Web of Science Core Collection, without any restriction of language or publication year. The number of periodicals with the most published articles in this theme, most published authors and keywords were mapped; other metrics were also evaluated such as the countries that have more research on the subject and the period in which there were more publications on the subject. During the knowledge mapping, the most frequent experimental designs were analyzed, type of saliva collection, stage of caries disease, evaluated oxidative parameters were retrieved and analyzed from each manuscript. Between the 43 selected articles, the *Journal of Clinical Pediatric Dentistry* was the periodical appearing the most with 4 published articles. The authors who published the most were Celec, P., Tothova, L., Hegde, A.M., Shetty, S., Antoniali, C., and Pessan, JP with three articles each, and a total of 180 keywords representing the evolution of the theme. India and Asia were found to be the country and continent with most publications, respectively. Most articles collected non-stimulated total saliva, with total antioxidant capacity being the parameter most often evaluated. The type of study that appeared the most was cross-sectional studies, and articles published in the period of 2017–2022 were the most frequent. Studies show that dental caries can be associated to the changes in salivary oxidative biochemistry with an increase in lipid peroxidation, a biomarker of oxidative damage, and an increase in antioxidant capacity in chronic caries, in response to cariogenic challenge. Some studies evidence the reduction of lipid peroxidation after treatment of the carious lesion. Our findings reveal worldwide research trends, as well as a clearer knowledge of the evolution and future scenarios of this issue, also showing the mechanisms associating dental caries with changes in salivary oxidative biochemical parameters are not clear.

## 1. Introduction

Oxidative stress is caused by an imbalance between the production of free radicals and non-radical species, such as reactive oxygen species (ROS), and the activity of enzymatic and non-enzymatic antioxidant systems, which are robust defense mechanisms against oxidative damage [1,2,3]. Therefore, pro-oxidant species can cause damage to cells and tissue by damaging lipids, proteins, enzymes, and DNA [4,5,6].

Oxidative stress is mainly assessed through the relationship between antioxidants (total antioxidant capacity, reduced glutathione, oxidized glutathione, glutathione peroxidase activity, superoxide dismutase, and catalase) and pro-oxidants (reactive species and levels of nitrates and nitrites), in addition to biomarkers of oxidative damage (lipid peroxidation and protein oxidation) [7,8,9,10,11,12].

By continuously soaking the teeth and oral mucosa, saliva acts as a cleaning solution, a lubricant, and buffer, and storage of calcium and phosphate [13]. These minerals are necessary for the remineralization of the first carious lesions in the oral cavity through the process of remineralization and demineralization of tooth enamel [13]. The biochemical constitution of saliva, which is supersaturated with the existing hydroxyapatite in tooth enamel, aids this action by limiting demineralization and boosting remineralization. Furthermore, saliva dilutes and neutralizes food acids and bacterial metabolism in the biofilm [13,14]. Saliva also plays an important role in protecting the oral cavity, and controlling dental caries due to its buffering effect and antioxidant capacity. The antioxidant of saliva establishes an equilibrium between free radicals, which play an important role in safeguarding the body [15,16]. In addition, salivary biomarkers of oxidative stress have been utilized to diagnose a variety of disorders in children’s oral cavities [17].

Saliva becomes the first line of defense against free radicals because it presents several antioxidant mechanisms (glutamate, ascorbic acid, uric acid, and melatonin), antioxidant enzymes (superoxide dismutase, catalase, and glutathione peroxidase) that will combat the negative effects of reactive oxygen and nitrogen species when found in excess in the oral cavity, beyond the levels necessary for physiological function [18]. Moreover, saliva has been shown to be a means of diagnosing oxidative stress, as these markers cause saliva to reflect changes that occur both in the oral cavity, as well as the pH balance and antioxidant capacity in the oral cavity [16]. It is also considered a fluid with a high capacity to detect molecules that can act as biomarkers for several oral diseases, such as periodontitis and dental caries [16]. Researchers observed a specific pattern of salivary antioxidant responses to oxidative stress in children with caries, with greater total antioxidant capacity (TAC) and superoxide dismutase (SOD) levels in caries-free children that were found lower malondialdehyde (MDA) levels [17,19].

In its turn, dental caries is the most frequent chronic oral disease and is considered a global oral health problem [20]. It is also considered an irreversible microbial disease that affects the hard tissue of teeth and is characterized by the destruction of the inorganic and organic portions of the tooth, leading to cavitation and possible tooth loss [14]. At an early stage of caries formation, signs of demineralization, such as white spotting, are observed in mineralized tooth tissues; however, disease formation occurs within the dental plaque that lies on the surface of the teeth [21]. Early diagnosis of caries is necessary to avoid tooth loss.

Some indicators of imbalance in the redox system were evidenced in the saliva of patients with dental caries, including changes in antioxidant and pro-oxidant parameters and biomarker of oxidative damage [16,22]. Changes in this system, characterized by increased levels of biomarker of oxidative damage and reduced antioxidant capacity are associated with the progression of dental caries [18]. Thus, this study mapped the overview of global scientific research on dental caries and salivary oxidative stress.

## 2. Materials and Methods

To perform this knowledge mapping we used bibliometric analysis tools, already described in previous studies of our group [23,24].

### 2.1. Search Strategy

A comprehensive search was performed by two independent examiners in the Web of Science Core Collection (WoS-CC) in November 2022, using the following search key: TS = (caries OR “Dental Decay” OR “Carious Lesion” OR “Dental White Spot” OR “tooth decay” OR “dental cavity” OR Cariology) AND TS = (Saliva).

### 2.2. Study Selection and Data Collection

Two independent authors selected the articles by reading the title, abstract and then reading the full text; search results were made, without restrictions on publication period and language. The criteria of choice were articles that focused on salivary oxidative stress and dental caries. Editorials, conference papers, letters, and commentaries, as well as studies that did not correspond to the specific theme, were excluded; if they met all eligibility criteria, they were included and in cases of disagreement between the reviewers, a third party resolved the disagreement.

After the selection of articles, TXT and Excel files were extracted from Web of Science. The TXT file was used to extract information such as the countries of the corresponding authors, keywords found in the papers, journals that had the most articles published on this theme, and publication density of the authors of the selected articles. The Excel file was used to extract the following data: authors, year of publication, number of citations, keywords, country, average per year, DOI, caries diagnostic, study design, age of the sample, countries, and continent of the corresponding author, abstract of the study. The number of citations of these articles in Google Scholar and Scopus was also collected to compare the number of citations with WoS-CC.

### 2.3. Data Analyses

Frequency analysis of descriptive measures and investigation of the authors’ collaboration network and keywords were performed using the VOSviewer software [25,26]. These terms were organized into clusters, and each cluster was represented by a color. The most important terms had larger circles, and the closely related terms were close together. In addition, the lines indicate the relationship between items, with a thicker line indicating a stronger connection. The MapChart tool (https://mapchart.net/ accessed on 30 November 2022) was used to illustrate the global distribution of articles selected.

### 2.4. Content Analysis

The selected articles were read in their entirety, seeking information about the experimental designs, the biochemical parameters investigated, the method used for saliva collection, the activity of the caries disease, the age of the patients, and the methods used for caries diagnosis, to observe the methodological patterns among the studies. The oxidative parameters that were analyzed by the articles were divided into three categories: antioxidants, pro-oxidants, and biomarkers of oxidative damage.

The study design was categorized as literature review, laboratory research (in vitro, in vivo, in situ, ex vivo), case reports/series, cross-sectional studies, case-control studies, cohort studies, longitudinal studies, clinical trials, and systematic reviews/meta-analyses [27].

## 3. Results

**Figure 1:**
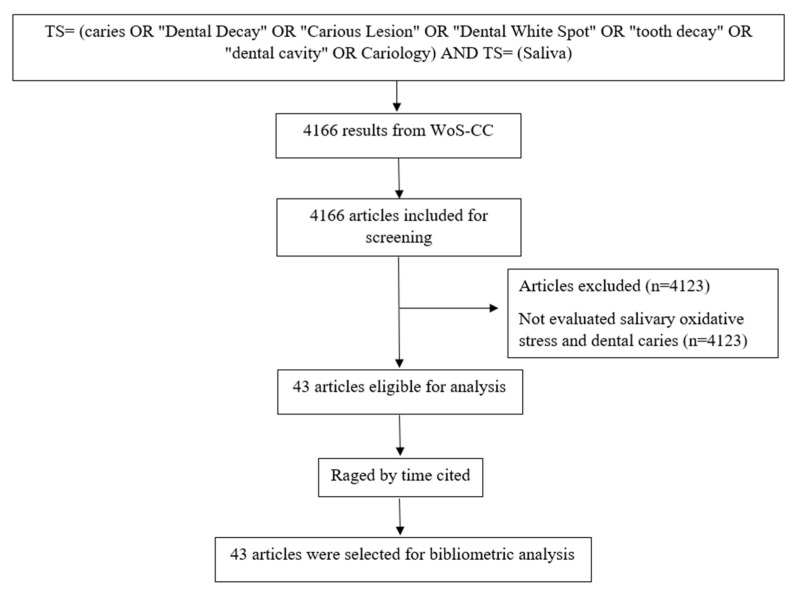
Flowchart of the search and article selection.

The search, in the WoS-CC, resulted in 4166 articles. A total of 43 articles were selected after reading the title, abstract, and full text (in case of doubt) (Figure 1) and 4123 were excluded.

The oldest study was conducted in 2005 [28]. The most recent studies from 2022 sought to group studies that evaluated salivary oxidative biochemical parameters and association with dental caries in children [29] and in children and adolescents [30] (Table 1), The period with the most published articles was between 2017 and 2022 (*n* = 23) (Table 2).

**Figure 2:**
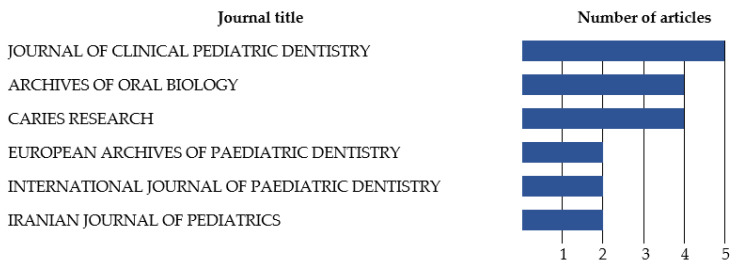
Journals that published at least two articles on dental caries and salivary oxidative stress.

The journal that published the most articles that related dental caries with salivary oxidative stress was the *Journal of Clinical Pediatric Dentistry* with a total of 5 articles out of 43, followed by the *Archives of Oral Biology* and *Caries Research* with 4 articles each (Figure 2).

**Figure 3:**
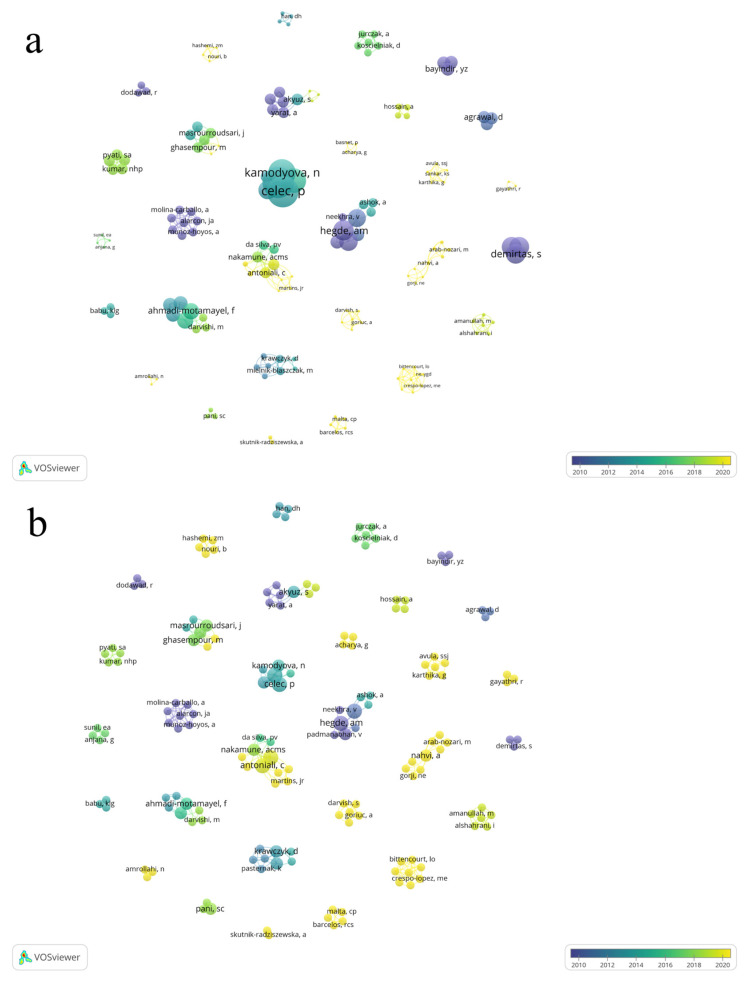
Co-authorship overlay visualization of the 43 articles showing the collaboration of the authors of the selected articles about the relation of dental caries and salivary oxidative stress. The total of 164 author form 30 clusters. The overlay visualization, the lines connecting authors the size of node denote number of articles (**a**) and number of citations (**b**), respectively.

The total number of authors was 164, of which 144 published only one article, 14 published two articles, and six published three articles. The largest contributions regarding the number of published articles were from the authors Celec, P., Tothova, L., Hegde, A.M., Shetty, S., Antoniali, C., and Pessan, JP, each with three published articles (Figure 3a).

**Figure 4:**
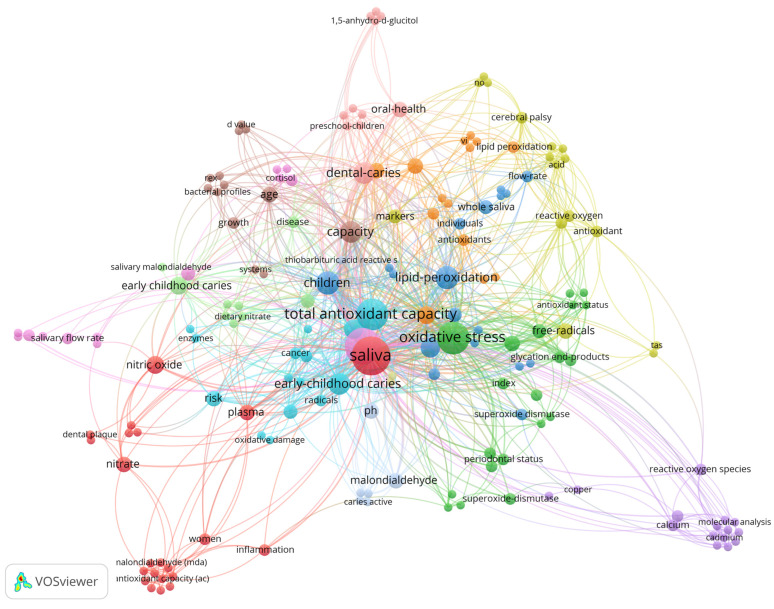
Network of co-occurrence of all keywords (author and keyword plus). The 180 keywords form 14 clusters. The size of the node represents the frequency of the keyword, with larger nodes indicating higher frequency.

The most cited authors were Celec, P. (166 citations) and Tothova, L. (166 citations), followed by Kamodyova, N. (131 citations), and Cervenka, T. (104 citations) (Figure 4).

A total of 180 keywords were identified and saliva (*n* = 27; 250 citations) is the most used and most cited keyword, followed by dental caries (*n* = 24; 201 citations), oxidative stress (*n* = 19; 183 citations), and total antioxidant capacity (*n* = 18; 154 citations). The distribution of the keywords is shown in Figure 4. The size of the node indicates the frequency of the keyword, the larger the node, the higher the frequency. The thickness of the edge is related to the closeness of the interactions between the two nodes. Note that the color of the node indicates the cluster to which the keyword belongs.

**Figure 5:**
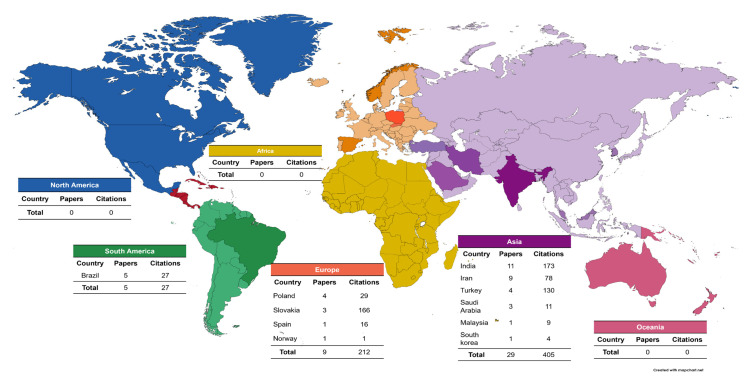
Geographical distribution of the selected articles.

The country with the highest number of publications was India, with a total of 11 published articles and a total of 173 citations. Asia had the most publications on caries and salivary oxidative stress with 29 articles and a total of 405 citations; South America published 5 articles; North America, Africa, and Oceania had no articles published in the area (Figure 5).

Among the study types, most were cross-sectional studies (*n* = 19), as well as case-control articles (*n* = 14), literature review (*n* = 4), systematic review (*n* = 4), experimental in vivo (*n* = 1) and longitudinal studies (*n* = 1) (Table 3).

The most cited article among the 43 selected articles was [18], which is a literature review that aims to gather information on the most frequently used salivary biochemical parameters and analyzes these markers in individuals with dental caries (Table 1).

The most performed form of saliva collection among the articles, for stress analysis, was unstimulated saliva, only two articles used the collection method through stimulated saliva [33,54]. Regarding the evaluation of dental caries, most of the articles did not evaluate according to the depth of caries, evaluating only the number of decayed teeth (Table 3).

Several oxidative parameters were evaluated in saliva, with variation in the evaluation method, and among the primary studies, the most evaluated salivary biochemical parameter was TAC (*n* = 22 articles) which was evaluated by six different methods, followed by LPO (*n* = 12 articles) which was evaluated in two different ways, the most common being the quantification of reactive substances to thiobarbituric acid, while the direct quantification of malondialdehyde was evaluated only in one study (Table 4).

## 4. Discussion

This study evidenced the scarce knowledge production on the association between dental caries with salivary oxidative stress in the literature. Among the 43 articles included, we obtained a total of 644 citations. The article that obtained the highest number of citations (104 citations) was a literature review, which comprehensively showed the relationship between saliva oxidative biochemical parameters and oral diseases, including caries in children [18]. In addition, most articles have evaluated TAC as the main parameter of biochemical changes in the saliva of patients with dental caries, and parameters such as glutathione, superoxide dismutase, uric acid, catalase, lipid peroxidation, and nitric oxide [7,18,38].

In information science, there are three empirical laws that are used to group empirical relations, namely Bradford’s law of bibliographic dispersion, which shows the relation between the most cited journals, Lotka’s law of scientific productivity, which shows the relation between the most cited authors, and finally, Zipf’s law of word frequency, which shows the relation between the keywords that appear most in bibliometric articles [65]. In our bibliometric review, we used these laws as a tripe, evaluating the metrics most used keywords, journals that published the most, and authors that published the most. When it comes to Bradford’s law, in our study we were able to verify six periodicals that had two or more articles published, and the Journal of Clinical Pediatric Dentistry had the most articles on this subject with a total of five articles.

Lotka’s law cites the importance of observing the network of collaboration among authors who research a given topic. In our study, when we observe the network of authors, we see that 164 participated in the 43 studies included and that a large part of these authors had only one publication in the area. Some authors were highlighted by being in three articles, authors Celec, P., Tothova, L., Hegde, A.M., Shetty, S., Antoniali, C., and Pessan, JP, who were the authors who most contributed to research on dental caries and salivary oxidative stress.

Following Zipf’s law, we analyzed the keywords that appeared most; saliva appeared most often, followed by dental caries, oxidative stress, and total antioxidant capacity. Saliva is a fluid present in the oral cavity responsible for cleaning the teeth and oral mucosa, buffering capacity, pH control, and lubrication, and acts as a reservoir of calcium and phosphate [13] It is a fluid with a high ability to detect biomarkers of various diseases of the oral cavity such as dental caries [13,16]. The included studies showed that any change in the oral cavity ends up unbalancing the levels of biomarkers, as in the case of salivary oxidative stress biomarkers, which in some studies were shown to be altered in the presence of dental caries, for example, total antioxidant capacity and superoxide dismutase were higher in dental caries groups [17,19]

One of the metrics evaluated was the number of citations. The number of citations of an article indicates what a particular study has managed to achieve; in principle, a highly cited article is seen as a watershed moment and may thus have a significant impact on research and practice [66]. In our study, no article reached several citations above 400, with the maximum combined citations of all articles being 644 citations, which suggests that this is not yet a widely studied subject.

When analyzing the country and continent of origin of the articles, Asia was the continent that had the most articles published within the theme of our study; it is possible to observe that the theme is still a little debated since we found only 43 published articles on this subject. It was also possible to observe that no article was retrieved from North America with this theme among the articles selected in the WoS-CC, which is interesting because the USA has the main research centers worldwide with the largest amount of funding for their research [67]. Thus, this may show that this theme is not yet a priority among the main research centers when analyzing the scientific production in WoS, even though saliva is proving to be a diagnostic medium for diseases, such as dental caries.

Dental caries is a multifactorial disease that affects the mineralized tissue of teeth through the metabolism of sugar by bacteria that produce acids that degrade these mineral tissues, altering the natural process of demineralization and remineralization that occurs in the oral cavity [20]. Early detection of dental caries is critical for less intrusive and productive treatment [68]. Thus, based on the International Caries Detection and Assessment System (ICDAS) and the International Caries Classification and Management System (ICCMS TM) procedures contained in the included publications, dentists now have a guide to estimate the risk of caries appropriately. Clinical practice is more successful when knowledge is shared with other experts [69]. As shown by [17], biomarkers of oxidative damage and antioxidants are altered depending on the stage of caries with increased TAC and SOD levels in this group when compared to caries-free children, in an environment where MDA is decreased.

Antioxidant capacity reflects the sum of the effects of all antioxidants, more specifically non-enzymatic antioxidants [17]. In saliva, antioxidant defense is composed of enzymes such as peroxidase, catalase, superoxide dismutase, and glutathione peroxidase, in addition to small molecules such as uric acid, and vitamin E [17,53]. Some studies showed that the level of antioxidants in dental caries children was higher when compared to the control group with no dental caries [17,45,53].

When it comes to antioxidant defense, in this study we found the keyword total antioxidant capacity to be one of the most frequently appearing and the most frequent biochemical parameter in most articles (evaluated in 27 articles). Total antioxidant capacity is the most used parameter because of its rapidity and low cost, and it provides an overview of all the antioxidants [9]. This parameter shows the combined effect of all antioxidants present in plasma and body fluids. However, due to its generalist aspect, total antioxidant capacity has some limitations, as it provides a limited answer about antioxidant defense mechanisms, thus not showing individually how each antioxidant agent acts in the face of increased production of reactive species [9].

The methods used to measure total antioxidant capacity may be sensitive to different types of antioxidants and performed by different methods, making the data between experiments ultimately not comparable [9]. This study identified five different methods for evaluating antioxidant capacity among the selected articles: according to the neutralization capacity of the ABTS radical [7,22,33,50,53,57,64], the capacity to reduce iron ions (FRAP) [7,17,18,19,40,44,48,52,55,56], to reduce phosphomolybdenum [49,58] the capacity to inhibit lipid peroxidation [1,7,59,60] and the capacity to inhibit ORAC [45] and the inhibition of crocin bleaching [62].

Most of the studies included in this review evaluated antioxidant capacity and/or antioxidants; to evaluate the redox system imbalance, malondialdehyde was measured directly or indirectly by measuring thiobarbituric acid-reactive substances, which were conducted in children and/or adolescents. Caries disease increases the levels of malondialdehyde and/or thiobarbituric acid reactive substances, thus demonstrating an association between caries and lipid peroxidation. Moreover, there was an increase in the activity of antioxidants, such as glutathione, glutathione peroxidase, nitric oxide, and vitamin C, as well as an increase in total protein levels. In this sense, the studies evaluated showed an association between caries and the increase in antioxidant capacity evaluated in saliva.

The levels between pro-oxidants and antioxidants end up changing according to the age of the individual. Araujo et al. (2020) [17] showed in their study that there was a change in the levels of total antioxidant capacity depending on the age of the individual: children showed higher total antioxidant capacity compared to that adolescents. Another study by Salman et al. (2021) [32] showed when only adolescents were isolated, a significant decrease in total antioxidant capacity was observed in the dental caries group, showing that antioxidant capacity may vary with age.

We can observe in the articles found, that most of them evaluated caries in children, and few studies were found that had evaluated adult patients. This may be due to the fact that in children it is easier to limit the influence of factors affecting the level of antioxidants, such as smoking, production factors, intake of medicines, and the presence of chronic diseases [70].

When it comes to pro-oxidants, malonaldehyde was the main pro-oxidant biochemical parameter evaluated among the included articles. Malonaldehyde is the most widely used method to indicate damage to biomolecules and can be performed directly or indirectly by testing substances reactive to thiobarbituric acid; the latter method is commonly used in screening procedures because it is a fast and low-cost method [17]. In this aspect, the selected studies showed a negative association between caries and the levels of malonaldehyde or thiobarbituric acid reactive substances.

We observed the great heterogeneity between the articles, which makes it difficult to compare the results since the studies used different ages (albeit in approximate age groups) and different stages of caries disease. Celecová et al. 2012 [55] showed that the effect of age should be considered in the evaluation of salivary markers of oxidative stress in relation to oral health, as it is a predictor for the variability of the analyzed markers.

Another important aspect is the specificity of each method used for evaluating the antioxidant capacity, in addition to this, studies showed values corrected by protein levels, while others express the results in different measures: gross value or by the percentage of the control, some analyses were performed by absorbance, others by fluorimetry, making the comparison between studies sensitive.

Our findings show the need for establishing standard methods and parameters for oral biology analysis in the field of oxidative stress. Differently from periodontology, cariology does not present a robust involvement in oxidative biochemistry fields, which can be visualized by the results described here. The purpose of this review with bibliometric approaches is broader than listing the evidence from peer-reviewed studies; this review brought the fragilities of this field, the research trends, and new perspectives in the field.

Oxidative stress must be understood as more than a biochemical process that may affect biological structures such as cells and tissues; it is also a chemical process that can oxidize other molecules available. Based on that, what are the impacts of oxidative stress on restorative products and techniques? What are the effects of salivary oxidative stress on the enamel quality and demineralization/remineralization process? In the case of salivary oxidative stress being associated with dental caries, is it reasonable to use antioxidant products? These and several other questions are raised after the complete reading and interpretation of our results, contributing to the scientific knowledge production in the field.

## 5. Conclusions

Our investigation demonstrated that the relationship between salivary oxidative stress and dental caries remains understudied, with the observation that only a few countries are studying this theme, with few studies examining the relationship between these disorders. Although there are some studies that revealed a link between caries and oxidative stress, more research is needed to accurately observe the interaction of salivary oxidative stress with dental caries and clarify the mechanism behind it. 

## Figures and Tables

**Table 1:** The articles selected about the relationship between dental caries and salivary oxidative stress.

Authors/Year	Abstract	DOI	Number of Citations		
			WoS-CC (Average Per Year ^a^)	Google Scholar	Scopus
de Sousa Né et al., 2022 [30]	A systematic review that sought to evaluate the association between biochemical parameters of salivary oxidative stress and dental caries.	10.3390/metabo12090858	0 (0.00)	0	0
Martins et al., 2022 [29]	A systematic review assessed the relationship between salivary biomarkers of oxidative stress and dental caries in children.	10.1016/j.archoralbio.2022.105432	1 (1.00)	1	1
Aliakbarpour et al., 2021 [31]	Compared children with various levels of early childhood caries to children without caries to evaluate thiobarbituric acid reactive substances as an indicator of lipid peroxidation, total protein, and pH of saliva.	10.1007/s40368-021-00672-9	1 (0.50)	1	1
Salman et al., 2021 [32]	Evaluated the association between oral dental caries and periodontal issues in a pediatric population and markers of oxidative stress.	10.3390/antiox10101540	1 (0.50)	1	1
Wagle et al., 2021 [33]	Search into the long-term changes in salivary nitric oxide, oxidative stress, and antioxidant capacity during pregnancy and correlate them with different stages of streptococcus mutans and lactobacillus colonization.	10.3390/ijerph18179330	1 (0.50)	3	1
Amrollahi et al., 2021 [34]	In this study, salivary malondialdehyde levels were compared between children who had early childhood caries and those who did not.	10.5812/ijp.113824	1 (0.50)	1	1
Karthika et al., 2021 [35]	Compared the levels of glutathione peroxidase and tocopherol with the incidence of dental caries in a sample of school-age children.	10.7860/JCDR/2021/47199.14986	1 (0.50)	1	*
Gorji et al., 2021 [36]	This meta-analysis study ascertains the association between salivary nitrous oxide concentration and pediatric dental caries.	10.5812/ijp.107050	0 (0.00)	2	1
Ravikumar et al., 2021 [37]	Measured the amounts of malondialdehyde in the saliva and to compare them to those of the three groups of kids who had different types of caries.	10.9734/JPRI/2021/v33i42A32395	1 (0.50)	*	*
Vahabzadeh et al., 2020 [38]	Assessed the relationship between various degrees of pediatric dental caries and salivary enzymatic antioxidant activity.	10.17219/dmp/126179	1 (0.33)	3	1
Skutnik-Radziszewska et al., 2020 [39]	A review of the literature to address the role of salivary redox biomarkers during caries and periodontal disease.	10.3390/app10186240	4 (1.33)	4	4
Shaki et al., 2020 [40]	Compared some of the chemical features of saliva, such as total antioxidant capacity, total protein, pH, and nitric oxide level, in children who were caries free and caries active.	10.22038/ijp.2019.42952.3598	3 (1.00)	4	*
Malta et al., 2021 [41]	Search how cerebral palsy and dental caries affected dental plaque index, salivary parameters, and oxidative stress in children and adolescents.	10.1007/s40368-020-00509-x	2 (0.67)	5	3
Araujo et al., 2020 [17]	Assessed the effect of dental caries severity (as determined by the ICCMS (TM) criteria) on the levels of oxidative stress biomarkers in children’s saliva.	10.1155/2020/3695683	6 (2.00)	16	7
Aksit-Bicak et al., 2019 [42]	Measured and compared the salivary nitric oxide levels of healthy dyspeptic and non-dyspeptic youngsters, as well as to evaluate its relationship to dental caries.	10.1186/s12903-018-0707-z	2 (0.50)	6	4
Syed et al., 2019 [43]	Assessed the relationship between children’s caries risk and salivary 1,5-anhydroglucitol, retinol, ascorbic acid, and tocopherol.	10.1155/2019/4503450	3 (0.75)	4	4
Rahman et al., 2019 [44]	A review of the literature to address the role of zinc and metallothionein in the development and progression of dental caries.	10.1007/s12011-018-1369-z	9 (2.25)	10	7
Alanazi et al., 2018 [45]	Total antioxidant capacity levels in children with severe early childhood caries were measured before and after dental therapy, and the results were compared to those of caries-free children.	10.1016/j.archoralbio.2018.08.002	5 (1.00)	11	4
Pani, 2018 [46]	Comprehensively examine the literature and do a meta-analysis on the relationship between pediatric dental caries and total antioxidant capacity.	10.4103/jispcd.JISPCD_203_18	3 (0.60)	7	*
Pyati et al., 2018 [1]	Measured and compared the levels of salivary flow, pH, buffer capacity, total protein, malondialdehyde and total antioxidant capacity between active and caries-free children.	10.17796/1053-4625-42.6.7	25 (5.00)	67	24
Ahmadi-Motamayel et al., 2018 [7]	Assessed salivary and serum total antioxidant capacity and malondialdehyde levels in dental caries patients.	10.1159/000488213	13 (2.60)	25	15
Mohammed et al., 2017 [47]	A literature review that shows the use of salivary biomarkers to diagnostic a lot of oral diseases including dental caries.	10.5005/jp-journals-10037-1093	0 (0.00)	2	*
Jurczak et al., 2017 [48]	Assessed the antioxidant barrier in the saliva of children with caries and how it affects the colonization of cariogenic bacteria.	10.1080/13510002.2017.1301625	12 (2.00)	23	12
da Silva et al., 2016 [19]	Evaluated oxidative stress levels and enzymatic and non-enzymatic antioxidant systems in the saliva of children with severe early childhood caries.	10.1016/j.archoralbio.2016.06.003	18 (2.57)	38	19
Tóthová et al., 2015 [18]	A literature review that sought to evaluate the salivary markers of oxidative stress in oral diseases, among them dental caries.	10.3389/fcimb.2015.00073	104 (13.00)	195	105
Subramaniam et al., 2014 [49]	Sought to assess total antioxidant capacity, nitric oxide, and sialic acid levels in the saliva of cerebral palsied toddlers.	10.17796/jcpd.38.3.tv26g158q7343287	12 (1.33)	30	14
Krawczyk et al., 2014 [50]	Examined the total antioxidant status in stimulated and unstimulated saliva in usually healthy non-smokers aged 15 to 17 in relation to the frequency of active carious lesions.	10.5604/12321966.1120605	5 (0.56)	17	6
Hegde et al., 2014 [51]	Evaluated superoxide dismutase activity, copper and zinc levels in the saliva of subjects with and without caries.	10.1159/000355580	18 (2.00)	43	22
Mahjoub et al., 2014 [52]	Compared the levels of total antioxidant capacity in the total unstimulated saliva of children with severe early childhood caries and caries-free children.	10.1159/000355581	21 (2.33)	45	21
Ahmadi-Motamayel et al., 2013 [53]	Evaluated the relationship between total antioxidant capacity of saliva and dental caries.	10.4317/medoral.18762	37 (3.70)	93	45
Han et al., 2013 [54]	Examine the relationship between salivary glutathione and tooth caries and cariogenic microorganisms.	10.1016/j.archoralbio.2012.09.021	4 (0.40)	10	5
Celecová et al., 2013 [55]	Verified if salivary markers of oxidative stress are related to age and oral health in non-smoking adults, observing the relationship with diseases such as dental caries.	10.1111/jop.12008	27 (2.70)	44	30
Tóthová et al., 2013 [56]	Analyzed salivary markers of oxidative stress in relation to periodontal and dental status in children.	10.1155/2013/591765	35 (3.50)	78	38
Krawczyk et al., 2012 [22]	Seed if there was a link between total antioxidant status of unstimulated whole saliva, patients’ ages, oral hygiene level, and dental caries.	10.2478/v10039-012-0015-9	8 (0.73)	15	8
Kumar et al., 2011 [57]	Estimated the total antioxidant capacity in the unstimulated saliva of healthy children with and without severe early childhood caries and to correlate the individual total antioxidant capacity level with ceo-d score and age.	10.1111/j.1365-263X.2011.01154.x	28 (2.33)	75	30
Hegde et al., 2011 [58]	Evaluated the oral hygiene status, salivary characteristics, and dental caries experience in children with acute lymphoblastic leukemia.	10.17796/jcpd.35.3.u5kx28q33m760834	28 (2.33)	75	35
Preethi et al., 2010 [59]	Evaluated the relationship between saliva physicochemical properties such as flow rate, pH, buffer capacity, calcium, total protein, and total antioxidant capacity in children with and without caries.	10.1007/s12291-010-0062-6	12 (0.92)	57	17
Hegde et al., 2009 [60]	Evaluated the total antioxidant capacity of saliva and its relationship with early childhood caries and rampant caries.	10.17796/jcpd.33.3.c730518021m56077	34 (2.43)	83	38
Ozturk et al., 2008 [61]	Evaluated the association between oral and dental health in young adults and salivary glutathione, lipid peroxidation and sialic acid levels and carbonic anhydrase activity.	10.1590/S0100-879X2008005000048	25 (1.67)	58	33
Uberos et al., 2008 [62]	Evaluated the relationship between total antioxidant capacity of saliva and the presence of dental caries in deciduous and permanent teeth, in a group of children from the Sahara.	10.1038/sj.bdj.2008.520	16 (1.07)	57	24
Hegde et al., 2008 [63]	Determined the levels of nitric oxide in the saliva of children with rampant caries and early childhood caries.	10.17796/jcpd.32.4.4010kl5262687528	14 (0.93)	36	16
Tulunoglui et al., 2006 [64]	Evaluated the relationship between saliva physicochemical properties such as flow rate, buffer capacity, pH, calcium level, total protein, total antioxidant status and dental caries, age, and sex.	10.1111/j.1365-263X.2006.00733.x	72 (4.24)	202	92
Bayindir YZ et al., 2005 [28]	Determined the correlation of the antibacterial substance nitric oxide with dental caries.	10.1159/000083158	31 (1.72)	66	34

WoS-CC: Web of Science Core Collection; * article not indexed; ^a^ Average per year based on the ratio of the numbers of citations and the period since the year of publication up to November 2022.

**Table 2:** Publication period of the selected articles.

Characteristic	Number of Papers	Number of Citations
Publication period		
2005–2010	7	204
2011–2016	13	345
2017–2022	23	95

**Table 3:** Types of studies of the selected articles.

Characteristic	Number of Papers	Number of Citations
Study design		
Cross-sectional	19	352
Case–control	14	152
Literature review	4	117
Systematic review	4	4
Experimental in vivo	1	18
Longitudinal study	1	1

**Table 4:** Evaluated parameters of saliva collection, caries, and oxidative biochemistry salivary in original articles.

Authors/Year	Saliva Collection	Stages of Caries Disease	Age (Years)	Caries Criterion	Salivary Oxidative Biochemistry (Assessment Method)		
					Antioxidants	Pro-Oxidants	Biomarker of Oxidative Damage
Aliakbarpour et al., 2021 [31]	Unstimulated whole saliva	Severe early childhood caries	3–5	DMFS	-	-	LPO (TBARS)
Salman et al., 2021 [32]	Unstimulated whole saliva	At least five decayed tooth surfaces requiring restoration	3–18	Caries Index	TAC (not informed)	-	LPO (TBARS), AOPP
Wagle et al., 2021 [33]	Stimulated whole saliva	-	30.81 ± 4.32	Not informed	TAC (ABTS)	NO	LPO (TBARS)
Amrollahi et al., 2021 [34]	Unstimulated whole saliva	Early childhood caries	4–6	DMFT	-	-	LPO (MDA)
Karthika et al., 2021 [35]	Unstimulated whole saliva	At least five decayed tooth surfaces requiring restoration	6–12	DMFT	Vitamin E, GPx	-	-
Ravikumar et al., 2021 [37]	Unstimulated whole saliva	Severe early childhood caries	3–6	DMFS	-	-	LPO (TBARS)
Vahabzadeh et al., 2020 [38]	Unstimulated whole saliva	At least five decayed tooth surfaces requiring restoration	7–12	DMFT	SOD, CAT, GPx activity	-	-
Shaki et al., 2020 [40]	Unstimulated whole saliva	five decayed tooth surfaces requiring restoration	3–5	DMFT	TAC (FRAP)	NO	-
Malta et al., 2021 [41]	Unstimulated whole saliva	At least five decayed tooth surfaces requiring restoration	2–20	DMFT	GSH, Vitamin C	Levels of reactive species	LPO (TBARS)
Araujo et al., 2020 [17]	Unstimulated whole saliva	Severe early childhood caries	1–3	ICCMS™ index	SOD, UA, TAC (FRAP)	-	LPO (TBARS)
Aksit-Bicak et al., 2019 [42]	Unstimulated whole saliva	At least five decayed tooth surfaces requiring restoration	6–16	DMFT	-	NO	-
Syed et al., 2019 [43]	Unstimulated whole saliva	At least five decayed tooth surfaces requiring restoration	6–12	DMFT	Vitamin A, Vitamin C, Vitamin E	-	-
Alanazi et al., 2018 [45]	Unstimulated whole saliva	Severe early childhood caries	5.13 ± 0.79	Not informed	TAC (ORAC)	-	-
Pyati et al., 2018 [1]	Unstimulated whole saliva	At least five decayed tooth surfaces requiring restoration	6–12	DMFS	TAC (Capacity to inhibit lipid peroxidation)	-	LPO (TBARS)
Ahmadi-Motamayel et al., 2018 [7]	Unstimulated whole saliva	At least five decayed tooth surfaces requiring restoration	15–19	DMFT	TAC (FRAP)	-	LPO (TBARS)
Jurczak et al., 2017 [48]	Unstimulated whole saliva	Early childhood caries	2–5	ICDAS	GSH, GSSG, GSH/GSSG, TAC (FRAP)	-	-
da Silva et al., 2016 [19]	Unstimulated whole saliva	Severe early childhood caries	0–3	DMFS	SOD, UA, TAC (FRAP)	-	LPO (TBARS)
Subramaniam et al., 2014 [49]	Unstimulated whole saliva	At least five decayed tooth surfaces requiring restoration	7–12	DMFT	TAC (Reduction of phosphomolybdenum)	NO	-
Krawczyk et al., 2014 [50]	Unstimulated whole saliva	At least five decayed tooth surfaces requiring restoration	15–17	DMFT	TAC (ABTS)	-	-
Hegde et al., 2014 [51]	Unstimulated whole saliva	At least five decayed tooth surfaces requiring restoration	25–50	DMFT	SOD, copper, and zinc levels	-	-
Mahjoub et al., 2014 [52]	Unstimulated whole saliva	Severe early childhood caries	3–5	DMFS	TAC (FRAP)	-	-
Ahmadi-Motamayel et al., 2013 [53]	Unstimulated whole saliva	At least five decayed tooth surfaces	15–17	DMFT	TAC (ABTS)	-	-
Han et al., 2013 [54]	Stimulated whole saliva	At least five decayed tooth surfaces requiring restoration	6–14	DMFT	GSH, GSSG	-	-
Celecová et al., 2013 [55]	Unstimulated whole saliva	Lesion affecting the dentin	19–83	Modified oral index	TAC (FRAP)	-	LPO (TBARS), AOPP, AGE
Tóthová et al., 2013 [56]	Unstimulated whole saliva	Lesion affecting the dentin	4–18	Modified oral index	TAC (FRAP)	-	LPO (TBARS) AOPP, AGE
Krawczyk et al., 2012 [22]	Not informed	Not informed	16–23	DMFT and DMFS	TAC (ABTS)	-	-
Kumar et al., 2011 [57]	Unstimulated whole saliva	At least five decayed tooth surfaces requiring restoration	3–5	DMFT	TAC (ABTS)	-	-
Hegde et al., 2011 [58]	Unstimulated whole saliva	At least five decayed tooth surfaces requiring restoration	4–10	DMFT	TAC (Reduction of phosphomolybdenum)	-	-
Preethi et al., 2010 [59]	Unstimulated whole saliva	At least five decayed tooth surfaces requiring restoration	7–14	DMFS	TAC (Capacity to inhibit lipid peroxidation)	-	-
Hegde et al., 2009 [60]	Unstimulated whole saliva	Early Childhood Caries, and Rampant Caries	6–12	WHO Oral Assessment Form.	TAC (Capacity to inhibit lipid peroxidation)	-	-
Ozturk et al., 2008 [61]	Unstimulated whole saliva	At least five decayed tooth surfaces requiring restoration	19–25	DMFT	GSH	-	LPO (TBARS)
Uberos et al., 2008 [62]	Unstimulated whole saliva	At least five decayed tooth surfaces requiring restoration	4–14	DMFT	TAC (inhibition of crocin bleaching)	-	-
Hegde et al., 2008 [63]	Unstimulated whole saliva	Early childhood caries	6–12	DMFT	-	NO	-
Tulunoglui et al., 2006 [64]	Unstimulated whole saliva	At least five decayed tooth surfaces requiring restoration	7–15	DMFS	TAC (ABTS)	-	-
Bayindir et al., 2005 [28]	Unstimulated whole saliva	At least five decayed tooth surfaces requiring restoration	18–25	DMFT	-	NO	-

-: not assessed; DMFT: decayed, missing, filled teeth; DMFS: decayed, missing, filled surface; ABTS: 2,2′-Azino-di-[3-ethylbenzthiazoline sulphonate; ICDAS: International Caries Detection and Assessment System; ICCMS^TM^: International Caries Classification and Management System; FRAP: ferric reducing ability of power; TBARS: tiobarbituric acid reactive substances; AOPR: advanced oxidation protein products; AGE: advanced glycation end products; UA: uric acid; ORAC: oxygen radical absorbance capacity; SOD: superoxide dismutase; GSH: glutathione; GSSG: oxidized glutathione CAT: catalase; NO: nitric oxide; MDA: malondialdehyde; GPx: glutathione peroxidase.

## Data Availability

The data of this manuscript has been included in the main text of this article.
